# K_V_7 Channels Regulate Firing during Synaptic Integration in GABAergic Striatal Neurons

**DOI:** 10.1155/2015/472676

**Published:** 2015-05-31

**Authors:** M. Belén Pérez-Ramírez, Antonio Laville, Dagoberto Tapia, Mariana Duhne, Esther Lara-González, José Bargas, Elvira Galarraga

**Affiliations:** División de Neurociencias, Instituto de Fisiología Celular, Universidad Nacional Autónoma de México, 04510 México City, DF, Mexico

## Abstract

Striatal projection neurons (SPNs) process motor and cognitive information. Their activity is affected by Parkinson's disease, in which dopamine concentration is decreased and acetylcholine concentration is increased. Acetylcholine activates muscarinic receptors in SPNs. Its main source is the cholinergic interneuron that responds with a briefer latency than SPNs during a cortical command. Therefore, an important question is whether muscarinic G-protein coupled receptors and their signaling cascades are fast enough to intervene during synaptic responses to regulate synaptic integration and firing. One of the most known voltage dependent channels regulated by muscarinic receptors is the K_V_7/KCNQ channel. It is not known whether these channels regulate the integration of suprathreshold corticostriatal responses. Here, we study the impact of cholinergic muscarinic modulation on the synaptic response of SPNs by regulating K_V_7 channels. We found that K_V_7 channels regulate corticostriatal synaptic integration and that this modulation occurs in the dendritic/spines compartment. In contrast, it is negligible in the somatic compartment. This modulation occurs on sub- and suprathreshold responses and lasts during the whole duration of the responses, hundreds of milliseconds, greatly altering SPNs firing properties. This modulation affected the behavior of the striatal microcircuit.

## 1. Introduction

GABAergic striatal projection neurons (SPNs) and GABAergic and cholinergic interneurons are the target of corticostriatal afferents [1]. Acetylcholine (ACh) plays a role in the processes that modulate cortical inputs onto SPNs [2–6] since cholinergic interneurons respond earlier than SPNs after a cortical command. However, few electrophysiological studies have explored cholinergic modulation during synaptic suprathreshold responses, whose synaptic convergence and integration are the basis of SPNs firing during “down”- to “up”-states voltage transitions [1, 7–9]. Presynaptic M_2–4_ type receptors modulate glutamatergic afferents to the striatum [10–13] and muscarinic M_1_ and M_4_ postsynaptic receptors are expressed in SPNs [14–17] where an array of intrinsic voltage dependent channels are regulated by them: calcium activated potassium channels [18], inward rectifying channels [19], transient K^+^ channels [20], cationic and sodium channels [12, 21, 22], and calcium channels [18, 23–25]. Currents carried by many of these channels have been shown to participate in the regulation of SPNs firing properties, but very few have been tested during synaptic responses [26–31]. One question is whether muscarinic G-protein coupled receptors and associated signaling are fast enough to intervene during the whole duration of corticostriatal suprathreshold responses.

The channels that greatly modify the firing properties of SPNs are modulated by muscarinic receptors, and one of those channels are the M-channels (encoded by K_V_7.2–K_V_7.5/KCNQ2–KCNQ5 genes) controlled by membrane voltage. In spite of their relatively small contribution at the soma compartment, these channels modulate membrane potential from subthreshold to suprathreshold ranges and control input resistance, action potential threshold, and excitability [32–35]. Moreover, novel types of plasticity have been disclosed [36]. One hypothesis is that many of these actions can be manifested during corticostriatal responses and regulate firing properties during synaptic integration, but this has not been shown. Therefore, here, we investigated the role of K_V_7/M channels in the corticostriatal synaptic integration of GABAergic SPNs* in vitro* by using selective agonist and antagonist (retigabine and XE991, resp.). We found that current carried by K_V_7 channels and regulated by muscarinic receptors greatly modifies the firing properties of GABAergic projection neurons during suprathreshold responses. Moreover, this firing modulation affects the behavior of the whole striatal microcircuit [37].

## 2. Material and Methods

### 2.1. Slice Preparation

The protocols followed the National University of Mexico guide for the care and use of laboratory animals (CICUAL-EGP41-14) including minimizing the number of animals to achieve statistical significance and the avoidance of animal suffering. D_1_ and D_2_ dopamine receptor eGFP BAC transgenic mice were used, between postnatal days 20–35 (developed by the GENSAT). Wild mice and nonfluorescent cells of BAC-mice were also recorded to detect possible inconsistencies due to transgenes expression. The animals were anesthetized with ketamine/xylazine. Their brains were quickly removed and placed into ice cold bath saline containing (in mM): 126 NaCl, 3 KCl, 25 NaHCO_3_, 1 MgCl_2_, 2 CaCl_2_, 11 glucose, 300 mOsm/L, pH 7.4 with 95% O_2_, and 5% CO_2_. Hemispheres were separated and parasagittal corticostriatal slices (250–300 *μ*m thick) were cut using a vibratome and stored in oxygenated bath saline at room temperature. Recordings were carried out in the dorsal striatum. Stimulation was performed with concentric bipolar electrodes (tip = 50 *μ*m) located in the cortex, as previously described [1]. After recordings, neurons were injected with biocytin and merged with eGFP-positive visualization or else, immunoreacted for ChaT to observe on a confocal microscope as previously described [8].

### 2.2. Current Clamp Recordings

We recorded from sagittal brain slices of BAC D1 or 2 eGFP transgenic mice. Slices were submerged in an iced saline solution containing (in mM): 124 NaCl, 2.5 KCl, 1.3 MgCl_2_, 2 CaCl_2_, 26 NaHCO_3_, 1.2 NaH_2_PO_4_, and 15 glucose (pH = 7.4, 300 mOsm/L, saturated with 95% O_2_ and 5% CO_2_). They were left for equilibration in this saline at room temperature for about 1 h. Single slices were transferred to a submerged recording chamber and superfused continuously with oxygenated saline (2-3 mL/min). Current-clamp recordings were performed with the patch clamp technique in the whole cell configuration in SPNs from the dorsal striatum. The slices were visualized using infrared differential interference contrast (IR-DIC) microscopy with an upright microscope and a digital camera. Data acquisition used software designed in the LabVIEW environment (National Instruments, Austin TX). Patch pipettes (3–6 MΩ) were filled with internal saline containing (in mM): 115 KH_2_PO_4_, 2 MgCl_2_, 10 HEPES, 1.1 EGTA, 0.2 ATP, 0.2 GTP, and 5% biocytin (pH = 7.2; 285 mOsm/L). In some experiments perforated patch clamp microelectrodes were used. No substantial differences between these configurations and previous recordings obtained with intracellular recordings were noted. Internal solution containing (in mM): 150 KCl, 10 HEPES, and final pH 7.2 and 280 mOsm/L was used. Stock solution of amphotericin B (66 *μ*g/mL) in dimethyl sulfoxide was diluted in the perforated patch internal solution for a final concentration of 180 *μ*g/mL. Transmembrane current was monitored continuously by applying a 10–20 mV pulse, from a holding potential of −80 mV.

Corticostriatal suprathreshold responses were evoked and recorded by stimulating sensory-motor cortical areas with concentric bipolar electrodes (50 *μ*m at the tip; FHC, Bowdoinham, ME). The distance between recording and stimulating electrodes was about 1 mm. Synaptic responses were evoked by a series of current pulses of increasing intensities until eliciting suprathreshold responses, with or without the firing of repetitive action potentials [8]. The stimuli were produced by the program but controlled by an isolation unit (Digitimer Ltd., England). The membrane potential was held at about −80 mV (near the “down”-state or resting membrane potential; −81 ± 5 mV; *n* = 24) while polysynaptic corticostriatal responses, lasting hundreds of milliseconds [1, 8], were induced.

### 2.3. Voltage-Clamp Recordings

Synaptic currents were evoked with field stimulation. The field electrode was positioned in the cortex: a bipolar concentric tungsten electrode (50 *μ*m at the tip). Paired stimuli were used to test the interference of presynaptic muscarinic receptors (20 ms of interstimulus interval; 0.2–0.4 ms duration; 1–40 V delivered through the stimulating electrode; at a frequency of 0.1 Hz). These experiments were carried out in presence of bicuculline (10 *μ*M). Traces shown are the average of 2 min recordings (10 traces) taken once the amplitude had been stabilized in a given condition. A small hyperpolarizing voltage command (10 mV) was constantly given during the experiment to monitor input conductance.

### 2.4. Calcium Imaging

These methods have been described before [38]. Briefly, mice were transcardially perfused with an ice-cold solution containing (in mM): 234 sucrose, 2.5 KCl, 7 MgCl_2_, 0.4 CaCl_2_, 28 NaHCO_3_, 1.44 NaH_2_PO_4_, 7 glucose, 0.28 ascorbic acid, and 4.5 pyruvate (pH = 7.4 with NaOH, saturated with 95% O_2_-5% CO_2_) before decapitation. Slices were then obtained with the same procedure as above. They were incubated in the dark for 40 min with 6.5 *μ*M fluo-4 AM (Invitrogen, Life Technologies) and equilibrated with 95% O_2_-5% CO_2_. Slices were then superfused with control saline in a chamber located on the stage of an upright microscope equipped with a 20x water-immersion objective (Olympus XLUMPlanFI; Olympus America Inc.). Excitation at 565 nm was performed with a Lambda LS illuminator (Sutter Instruments, Novato, CA). Experiments were performed at room temperature. Images were acquired with a cooled digital camera (CoolSNAP K4, Photometrics; Roper Scientific, Tucson, AZ) at 100–250 ms/frame. Data acquisition software was also designed in the LabVIEW environment. The imaged field was 800 × 800 *μ*m. Short movies (180 s and 20 ms exposure) were taken at different pharmacological conditions. The same program performed preliminary image processing. All active neurons in a field were automatically identified and their mean fluorescence was measured as a function of time. Single pixel noise was discarded using a 5-pixel ratio mean filter. Calcium dependent fluorescence signals were computed as (*F*_*i*_ − *F*_*o*_/*F*_*o*_), where *F*_*i*_ is fluorescence intensity at any frame and *F*_*o*_ is resting fluorescence. Calcium signals elicited by action potentials were detected based on a threshold value given by the first time derivative of their calcium transients (2.5 SD of the noise) [38]. Calcium transients were signaled by dots in a raster plot where each row represented the activity of one neuron and the *x*-axis represents time. Summed activity was graphed below the raster plot in a form of histogram. 10,000 Monte Carlo simulations were used to find the significance of neurons being active together. In this way we could follow the activity of dozens of neurons with single cell resolution [38].

### 2.5. Materials and Drugs

For current clamp and calcium imaging recordings drugs were administered into the bath saline. Substances used were then added to the superfusate from thawed stock solutions. Muscarinic toxin mamba toxin 7 (MT-7) and the KCNQ agonist retigabine were obtained from Peptides International (Cat. number PMT-4340-s, Louisville, KY, USA). Muscarine, bicuculline, and biocytin were obtained from Sigma-Aldrich-RBI (St. Louis, MO, USA). KCNQ antagonist XE991 was obtained from Tocris (Bristol, UK).

### 2.6. Data Analysis

Digitized data was imported for analysis and graphing into commercial software (Origin 7, Microcal, Northampton, MA, USA; RIDD: rid_000069). Representative mean ± S.E.M. of the areas under synaptic responses was measured and compared. Paired or unpaired Student *t*-tests or one way ANOVA plus* post hoc* Bonferroni tests were mostly used upon repeated measurements (Systat 11, RRID: nlx_157643 and Graphpad Prism 5, RRID: rid_000081; San Jose CA, USA). Upon small samples, distribution-free statistics were also performed: Friedman or Kruskal-Wallis ANOVA tests with* post hoc* Dunnette's, Wilcoxon's or Mann-Whitney's tests (depending on paired or nonpaired samples) when comparing several treatments. *P* < 0.05 was used as significance threshold.

## 3. Results and Discussion

### 3.1. Muscarinic Actions on Corticostriatal Responses

The striatum is mainly composed of GABAergic neurons: about 90–95% are striatal projection neurons (SPNs) and about 5–10% are interneurons, most of them being GABAergic. In addition, this nucleus is extremely rich in acetylcholine (ACh), cholinergic receptors, and cholinergic interneurons [16, 17, 39–41]. Several classes of striatal interneurons activate slightly before or in correlation with SPNs following a cortical stimulus. A suprathreshold stimulus may activate SPNs directly and indirectly through the polysynaptic activation of interneurons and other SPNs [1, 42]. Besides activating glutamatergic and GABAergic receptors, polysynaptic responses last hundreds of milliseconds and include the activation of muscarinic receptors as well as several classes of intrinsic voltage dependent currents [24, 26, 43, 44]. This multisynaptic and convergent activation is one origin of “down”- to “up”-states voltage transitions [7].

**Figure 1:**
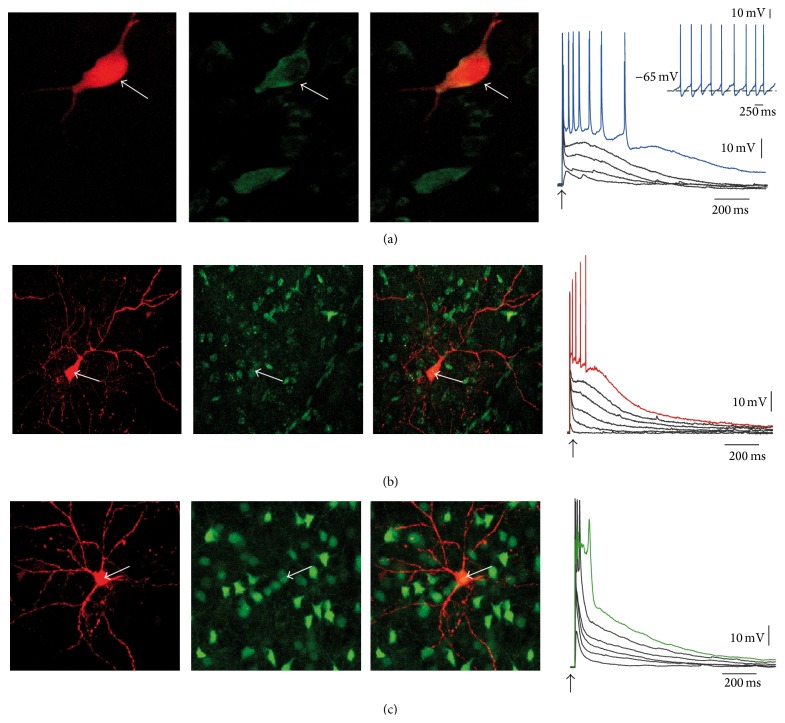
Identification of some striatal neurons responding to suprathreshold cortical stimulation. (a) Cholinergic interneuron (arrows): left: filled with biocytin-red-CY3; middle: immunocytochemistry for ChaT (green); right: merge. Voltage recordings correspond to synaptic responses to cortical stimulation of increasing strength (arrow). Blue trace corresponds to the strongest strength; note repetitive firing of action potentials. Inset: when holding current is zero these neurons tend to fire in a tonic fashion. (b) Left: a BAC-D_1_R-eGFP neuron injected with biocytin-red-CY3; middle: the neuron expresses GFP; right: merge. Voltage recordings correspond to synaptic responses of increasing strength (arrow). Red trace corresponds to the strongest strength; note a brief train of action potentials. (c) Left: a BAC-D_2_R-eGFP neuron injected with biocytin-red-CY3; middle: the neuron expresses GFP; right: merge. Voltage recordings correspond to synaptic responses of increasing strength (arrow). Green trace corresponds to the strongest strength; note a briefer train of action potentials and an autoregenerative propagated event.

Figure 1 shows typical firing modes of three double labeled and identified striatal neurons upon cortical stimulation: cholinergic (Figure 1(a)), a D_1_-receptor expressing direct pathway striatal projection neuron (dSPN) (Figure 1(b)), and a D_2_-receptor expressing indirect pathway striatal projection neuron (iSPN). It has been shown that cholinergic neurons respond with a slightly briefer latency than the responses of SPNs [1, 24]. In addition, continuous firing of cholinergic interneurons maintains a tonic level of ACh in the striatum [7, 24, 45], muscarinic M_1_ receptors are expressed in all SPNs, and K_V_7 channels have been shown to compose a minor but functionally important part of the intrinsic voltage gated currents that are present in all SPNs [34].

**Figure 2:**
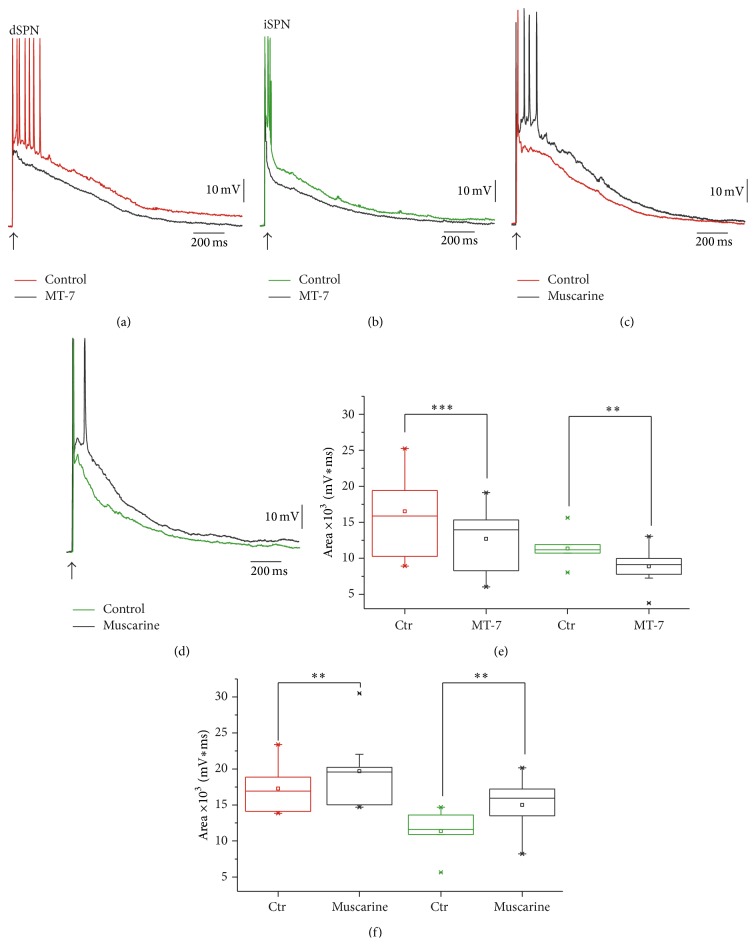
Suprathreshold responses of SPNs involve a G-protein coupled muscarinic component. (a), (b), Corticostriatal suprathreshold responses in a dSPN (a) and in an iSPN (b): 50 nM of the selective antagonist of muscarinic M_1_ class receptors, mamba toxin 7 (MT-7), reduced the amount of depolarization caused by the same stimulus in both neuron classes, indicating that endogenous ACh is necessary to reach these levels of depolarization during cortical stimulation. Colored traces: controls; black traces: during MT-7. ((c), (d)) Note that while activating with muscarine (1 *μ*M), the opposite actions are obtained. There is an enhancement of evoked depolarization, thus adding to the action of endogenous ACh. ((e), (f)) Tukey box plots compare the area under the synaptic response (mVms) in both classes of SPNs, for MT-7 and muscarine applications.

Here, we show evidence (Figure 2) that the response of SPNs to the same cortical stimulus is affected by activating muscarinic receptors. The same results can be obtained with perforated or nonperforated whole-cell recordings as well as with intracellular recordings [8]. Thus, Figures 2(a) and 2(b) show that the depolarization evoked by cortical stimulation was reduced in both classes of SPNs by blocking the activation of M_1_ receptors by the very selective mamba toxin 7 (50 nM MT-7) [46, 47]; indicating that G-protein coupled signaling activated by endogenous ACh was necessary to attain the level of depolarization to achieve repetitive firing [18]. After blockade of muscarinic M_1_ class receptors the area under the synaptic response of dSPNs decreased by 23% (from 16,510 ± 1,495 mVms to 12,690 ± 1,218 (mVms); ^*∗∗∗*^ *P* < 0.0005; *n* = 12; Figure 2(e)) and the same actions were revealed for iSPNs: MT-7 decreased the area under the synaptic response by 22% (from 11,360 ± 809 mVms to 8,891 ± 853 mVms; ^*∗∗*^ *P* < 0.0005; *n* = 9; Figure 2(e)). In both cases, firing was severely affected.

The reverse experiments are shown in Figures 2(c) and 2(d): muscarinic M_1_ class receptors were activated by using the agonist muscarine (1–10 *μ*M): in dSPNs the response implied a larger depolarization reflected by the area under the synaptic response that increased 14% (from 17,290 ± 1,155 mVms to 19,690 ± 1,811 mVms; ^*∗∗*^ *P* < 0.008; *n* = 8; Figure 2(f)). The synaptic response of iSPN also increased for the same stimulation intensity by 32% (from 11,360 ± 983 mVms to 15,010 ± 1,203 mVms; ^*∗∗*^ *P* < 0.002; *n* = 10; Figure 2(f)). Modulation lasted the whole duration of the responses and it does not appear to be saturated since muscarinic actions could add to the endogenous ACh actions. Apparently, the activation of muscarinic receptors may produce changes in passive properties (e.g., electrotonic decay and membrane resistance) in the membrane compartment where most synaptic inputs are generated (mostly secondary and tertiary dendrites) and on intrinsic currents that become activated during synaptic suprathreshold depolarization [24, 26] to explain the changes in amplitude of these responses.

Thus, previously, we have reported other muscarinic actions on the corticostriatal responses, for instance, the boosting of synaptic responses by facilitating Ca^2+^-currents. But these actions were due to muscarinic M_4_ class receptors that appeared to act only in dSPNs [24]. In addition, modulation of Ca^2+^-activated K^+^-currents shunts the trains of action potentials generated in iSPNs making them briefer than those generated in dSPNs [26]. Therefore, the responses now described are the first to affect both classes of SPNs in the same way and with the same relative magnitude, affecting their whole duration. The obvious candidates to explain these responses are the K_V_7/KCNQ channels classical effectors of muscarinic receptors, because it has been demonstrated that activation of muscarinic receptors closes K_V_7 channels in these cells [34]. Moreover, single-cell reverse transcriptase-PCR confirmed the expression of KCNQ_2,3,5_ mRNAs in SPNs, although their contribution to whole cell K^+^-current is relatively small [34]. Therefore, we next evaluated the action of these channels on the suprathreshold response.

### 3.2. K_V_7 Actions on Corticostriatal Responses in Both Classes of SPNs Are Similar

**Figure 3:**
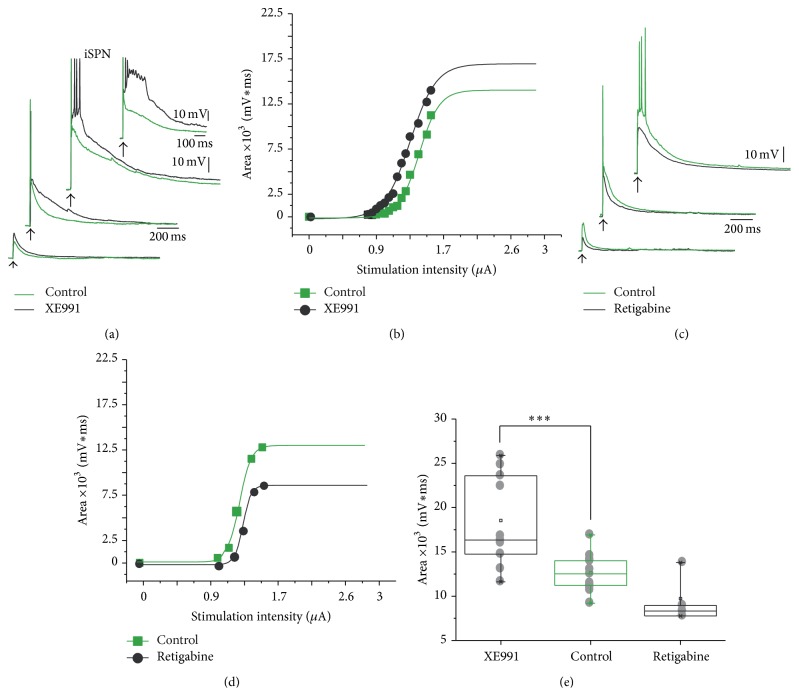
Corticostriatal responses of iSPNs during application of selective antagonist and agonist of K_V_7 channels. (a) After blocking K_V_7 channels with 10–20 *μ*M XE991 the corticostriatal responses of iSPNs increased. Note the enhancement for all stimulus strengths (green traces are the controls; black traces are recordings during XE991). Note the enhancement of regenerative responses at the highest strengths. (b) The whole intensity-response relationship, measured as the area under the responses was shifted to the left. (c) The reverse experiment: a K_V_7 channel opener 10–20 *μ*M, retigabine, had opposite actions: responses were decreased, showing that endogenous ACh has an action at all intensities of stimulation (control in red, retigabine in black). (d) Now intensity-response plot was shifted in an opposite direction (right). (e) Tukey box plots summarize these experiments.

**Figure 4:**
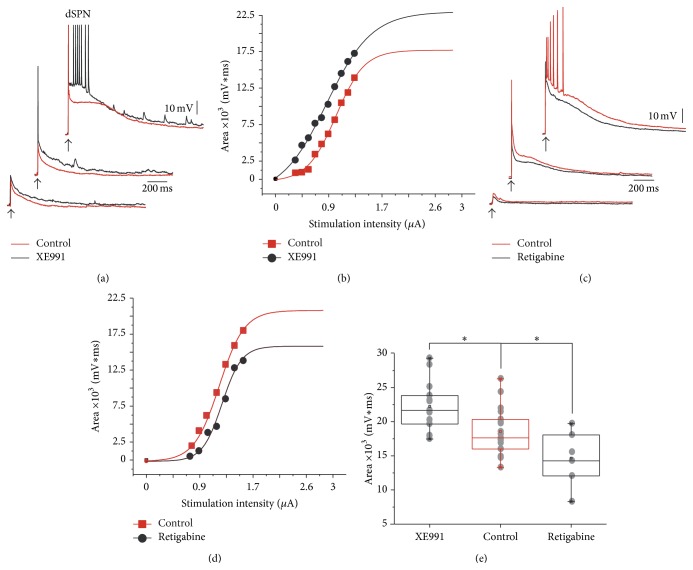
Corticostriatal responses of dSPNs during the application of selective antagonist and agonist of K_V_7 channels. (a) After blocking K_V_7 channels with 10–20 *μ*M XE991 the corticostriatal responses for all stimulus strengths increased (red traces are the controls; black traces are recordings during XE991). Note that dSPNs do not exhibit regenerative responses but do exhibit “spikelets” along the trace. (b) The whole intensity-response relationship, measured as the area under the responses, was shifted to the left. (c) The reverse experiment, a K_V_7 channel opener 10–20 *μ*M retigabine, had opposite actions: responses were decreased, showing that endogenous ACh has an action at all intensities of stimulation (control in red, retigabine in black). (d) The intensity-response plot was now shifted to the right. (e) Tukey box plots summarize these experiments.

To test the consequences of activating K_V_7 channels in both classes of SPNs we used very selective pharmacological tools (Figures 3 and 4). First, we tested the response of iSPNs after single pulses of increasing intensity to evoke subthreshold, threshold, and suprathreshold responses, before (green traces are control records) and during 10–20 *μ*M XE991, a K_V_7 channel antagonist. Similarly to the case of muscarinic M_1_ class receptor blockade, the closing of K_V_7 channels enhanced the evoked depolarization at all tested strengths of stimulation (black traces during XE991; Figure 3(a)). This is expected since XE991 is a K_V_7 channel blocker that decreases membrane conductance thus boosting synaptic responses. In the inset of Figure 3(a) it is shown that autoregenerative calcium potentials (*n* = 9 out of 11 neurons) could be evoked after XE991 facilitation of the synaptic response [8, 48, 49], suggesting that these channels are necessary to control this outcome.  Figure 3(b) shows that increases in the areas under the responses occurred at all intensities as seen at the soma, confirming that even at subthreshold responses muscarinic receptors are activated. On the other hand, the use of a channel agonist (opener), retigabine (10–20 *μ*M) had the opposite action: it reduced the responses at all intensities (Figure 3(c); green traces are the controls; black traces were recorded during retigabine) suggesting that membrane conductance in the region where synaptic inputs arrive is increased. The curve depicting the areas under the responses indicated that action occurred at all stimulus strengths. The statistical analysis of this sample of neurons used suprathreshold responses and is summarized in Figure 3(e). In iSPNs, XE991 increased the area under suprathreshold synaptic responses by 50% (from 10,210 ± 709 mVms to 15,360 ± 1,529 mVms; ^*∗∗∗*^ *P* < 0.001; *n* = 11). In contrast, retigabine reduced the area under the response to 9,712 ± 1387 mVms (*P* < 0.05; *n* = 6; using one way ANOVA with Dunnett's* pos hoc* test comparing XE991 with controls and retigabine with controls).

Similar experiments were performed in a sample of dSPNs. Synaptic responses of dSPN in presence of XE991 (10–20 *μ*M) enhanced the area under the synaptic response at all stimulus strengths, in particular during suprathreshold responses by 23% (from 15,440 ± 826 mVms to 18,930 ± 1,123 mVms; ^*∗∗*^ *P* < 0.0012; *n* = 13; Figures 4(a) and 4(b)). The K_V_7 agonist, retigabine (10–20 *μ*M), had opposite effects: area under suprathreshold responses decreased by 32% (from 16,910 ± 743 mVms to 11,370 ± 1,238 mVms; ^*∗*^ *P* < 0.031; *n* = 6; Figures 4(c) and 4(d)). Sample summary is illustrated in Figure 4(e): one way ANOVA with Dunnett's multiple comparison test indicated that differences between XE991 and retigabine with the controls were significant (^*∗*^ *P* < 0.05).

**Figure 5:**
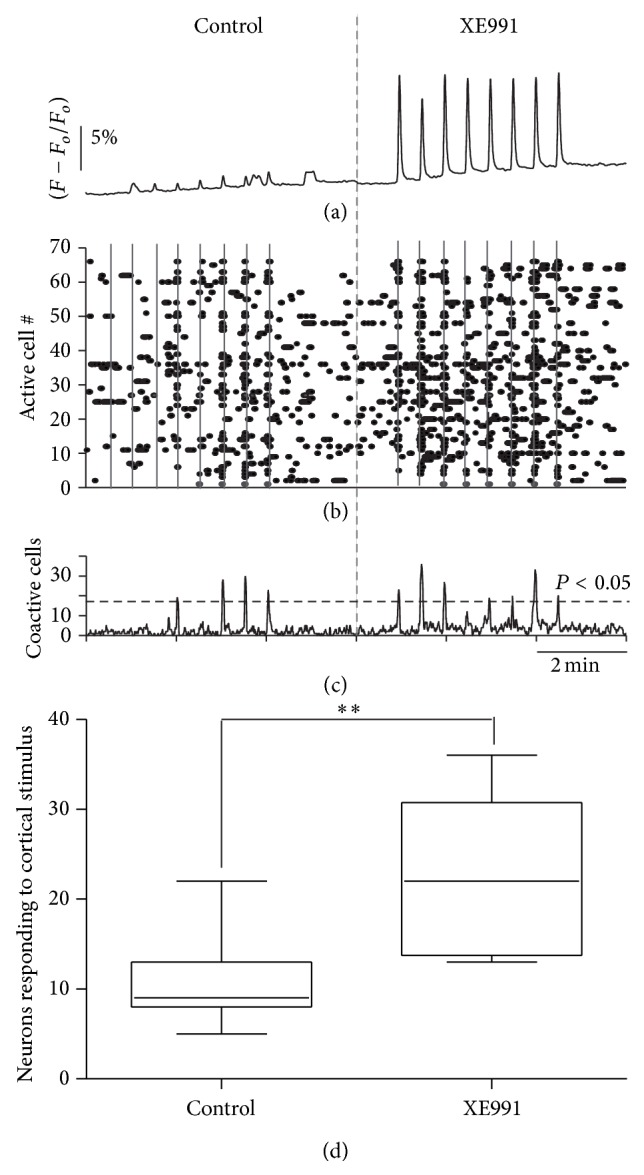
Activation of K_V_7 channels during corticostriatal responses in SPNs is a robust phenomenon that correlates with Ca^2+^ entry. (a) Example of a single neuron responding to cortical stimulus of increasing strengths that evoked intracellular Ca^2+^ transients before (control) and after addition of XE991 into the bath saline (XE991). Calcium indicator was fluo-4. There was an increase in evoked Ca^2+^ transients in the same neuron after 20 *μ*M XE991; it correlates to voltage responses and increase in firing (Figures 3 and 4). (b) Raster plot showing the same experiment but watching dozens of neurons simultaneously with single cell resolution: dots denote the activity of fluo-4 imaged neurons: each dot denotes an intracellular Ca^2+^ transient, *x*-axis is time, each row in the *y*-axis represents the Ca^2+^ transients (activity) of a single neuron, and grey columns indicate the times of cortical stimulus. Note less activity in the control side. (c) Histogram showing the summed activity of neurons above. When the stimulus coactivated a significant number of neurons in the same image frame it was denoted by a significant peak of synchronization (significance obtained with Monte Carlo simulations). There are more peaks of synchronization after XE991 (*n* = 6 slices from different animals). (d) Tukey box plots summarizing sample statistics.

During single recordings in cell-focused studies there are often sources of variation such as the position of the electrodes in each experiment, whether the recorded cell was a main target of the cortical afferents being activated, and the activity of the microcircuit itself when each neuron has a role in a reverberant type of activity [21]. Therefore, to reinforce the statistical value of this findings we performed experiments where the activity of several of neurons were recorded by means of Ca^2+^ imaging using fluo-4 [38].  Figure 5(a) illustrates one example of how cortically evoked intracellular Ca^2+^ transients augmented by the presence of XE991 in one cell [28].  Figure 5(b) shows a raster type of plot where dots represent intracellular Ca^2+^ transients as those in Figure 5(a) (cell activity), the *x*-axis denotes time, and each row of the *y*-axis represents activity of a single neuron. There was more evoked activity during XE991 than in the control (*n* = 6 slices from different animals). The histogram of Figure 5(c) illustrates the summed activity of neurons in the raster plot where each gray column denotes the times of stimulation. Cortical stimulus evoked more peaks of significant coactive neurons in the presence of XE991, suggesting that the action of the cortex was facilitated by activating more neurons when K_V_7 channels were blocked. Tukey plots in Figure 5(d) summarizes the statistics of this sample (^*∗∗*^ *P* < 0.02; Mann Whitney's *U* test).

The action mediated by K_V_7 channels in the suprathreshold responses (according to pharmacological tools) is the first one that affected both classes of neurons during the whole duration of their responses. These responses are the most similar to those shown for M_1_ receptors (Figure 2) in sharp contrast with previous reports of muscarinic involvement in the synaptic corticostriatal response of SPNs, while M_4_-receptor action is only present in dSPNs [24] and Ca^2+^-activated K^+^-currents act differentially in dSPNs and iSPNs [26]. It is known that M_1_ muscarinic receptors close K_V_7 channels through the phosphorylation of PIP_2_ [34]. And although differences reported for each type of SPNs remained (enhanced regenerative events in iSPNs; Figure 3(a); and more prolonged trains of spikes in dSPNs), the closing of K_V_7 channels, acting on both cell classes, augments the circuit activity after a cortical command.

### 3.3. K_V_7 Blockade Evokes Down- and Up-State Voltage Transitions

**Figure 6:**
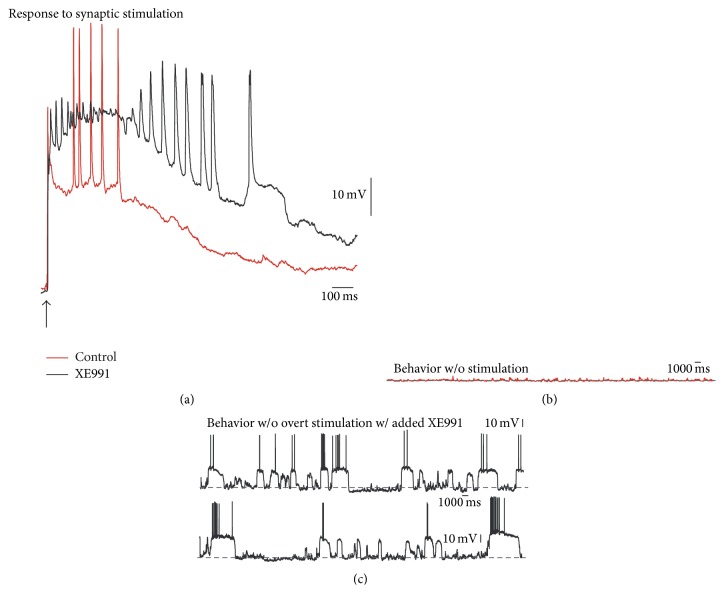
Blockade of K_V_7 channels evokes oscillatory up- and down-states in a dSPN. (a) A suprathreshold corticostriatal response after a single cortical stimulus before (red: control) and after addition of XE991 (20 *μ*M; black recording). Note increased depolarization with spike inactivation and a prolonged spike train. (b) During control (red trace) most dSPNs are silent. (c) After addition of XE991 (black traces), dSPNs exhibited down- to up-states transitions that lasted several hundred milliseconds to seconds in 23% of the recorded cells. These transitions could be observed for more than half an hour.

iSPNs have been posited as more excitable than dSPNs [50]. Surprisingly, however, K_V_7 blockade with XE991 evoked “down”- and “up”-state voltage transitions more readily in dSPNs than in iSPNs as recorded in whole cell.  Figure 6(a) shows that XE991 (10–20 *μ*M) enhanced the depolarization and duration of action potentials trains in dSPNs (red control; black with XE991). Commonly, these cells are silent without stimulation (Figure 5(b)). However, in the absence of any overt stimulus, addition of XE991 into the bath saline evoked transitions between “down”- and “up”-states (Figure 6(c)), many of them sustaining trains of action potentials. This behavior was observed in 23% of recorded cells (3 out of 13) and could be observed for up to 45 min. This oscillatory behavior was similar to that evoked with NMDA* in vitro* [51].

**Figure 7:**
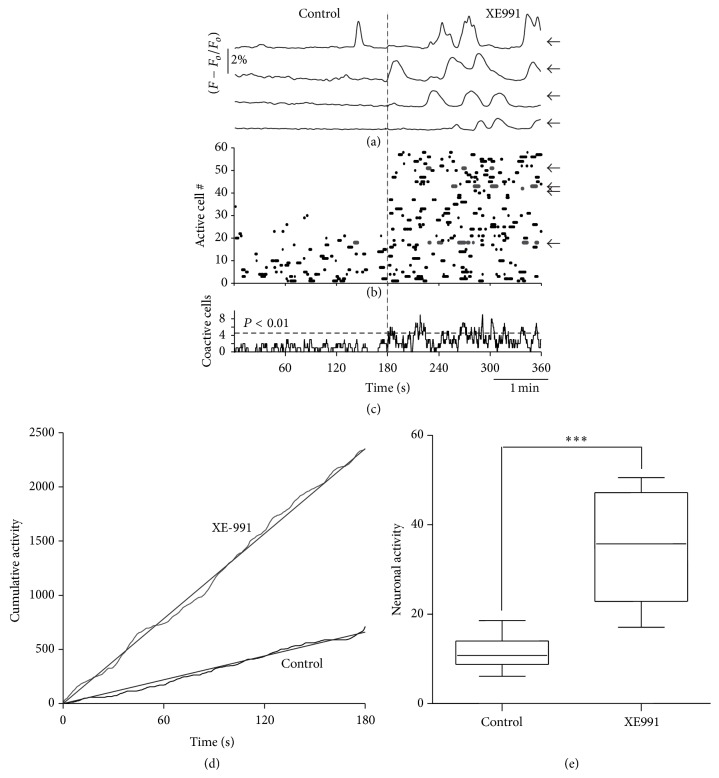
Oscillatory up- and down-states provoked by XE991 increased spontaneous microcircuit activity. (a) Spontaneous intracellular Ca^2+^ transients recorded in four different neurons, before (control) and after XE991 application (XE991) to the superfusion. (b) Raster plot as in Figure 5: there is less activity in the control than during XE991 application (neurons sorted in ascending order to signal the ones recruited after XE991). Arrows indicate the ones that exhibited the Ca^2+^ transients in (a). (c) Histogram with the summed neuronal activity frame by frame. There are no significant peaks of coactive neurons in the control but they appear after XE991 addition. (d) Cumulative neuronal activity is the area below the histogram in (c) summed all through the movie. Cumulative activity is significative after XE991. (e) Tukey box plots summarizing results from this sample of experiments showing that XE991 significantly increased microcircuit activity.

By recording in small samples of cells, one cannot be sure that this action of XE991 is significant for the microcircuit. Therefore, in Figure 7 we show a representative experiment with Ca^2+^ imaging and simultaneous recordings of a population of SPNs. Clearly, intracellular Ca^2+^ transients increased in several cells (Figure 7(a)), and the raster plot (as that in Figure 5 but without cortical stimulation; Figure 7(b)) shows more neurons active during XE991 (neurons were sorted in ascending order to separate spontaneously active neurons from those recruited after XE991). Histogram of summed activity shows significant peaks of coactive cells only after XE991 (Figure 7(c); *n* = 6 slices from different animals). Moreover, when the areas under the histogram are summed through time there is clearly more cumulative activity during XE991 (Figure 7(d); slope ± estimation errors: control: 12.96 ± 0.67; XE991: 32.58 ± 0.93; *P* < 0.001; *n* = 8). Total numbers of active cells were also significantly different (Figure 7(e); ^*∗∗∗*^ *P* < 0.003; Mann-Whitney's *U* test). To conclude, these experiments demonstrate that the closing of a single class of K^+^ channels, K_V_7, not only affects scattered cells but the behavior of the circuit as a whole, even if they comprise a small fraction of whole cell K^+^ current [34]. Analysis of the resultant circuit is out of the scope of the present work. But evidences that K_V_7 channels are involved in the control of correlated firing exist [35, 52]. These results suggest that the great increase in circuit activity during Parkinson's disease may be in part due to hypercholinergia [21].

### 3.4. Actions of K_V_7 Channels Are Postsynaptic

**Figure 8:**
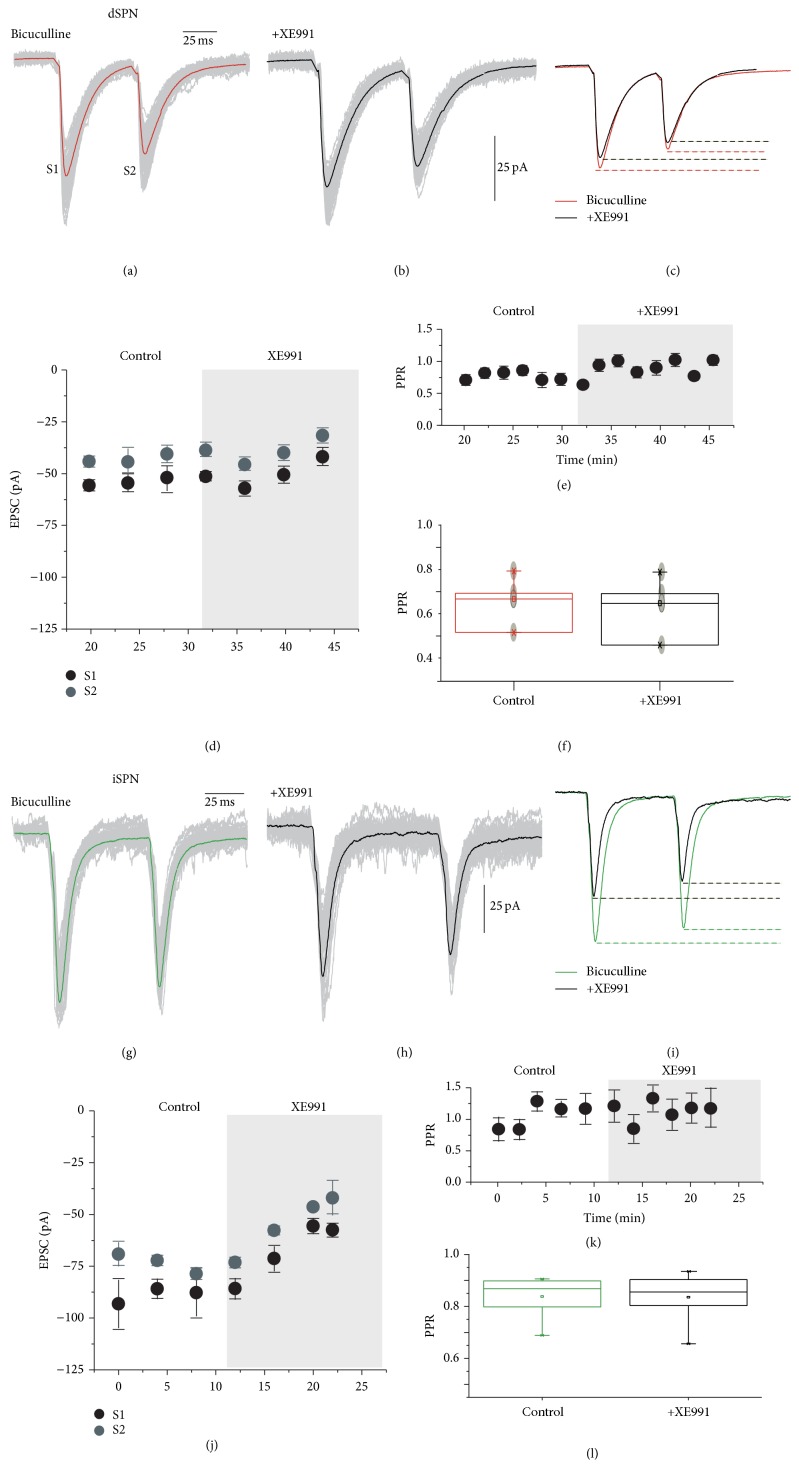
Blockade of K_V_7 channels is postsynaptic. (a) A pair of evoked excitatory postsynaptic currents (EPSCs) evoked from the cortex in a dSPN in the presence of bicuculline. (b) The decrease in conductance produced by XE991 produces a small decrease in EPSCs amplitude as detected at the soma. (c) Superimposition of (a) and (b). (d) Time course of amplitude changes in EPSC. (e) No significant change in the paired pulse ratio (PPR) was detected during the time of the experiment. (f) Tukey box plots showing that changes in PPR in the whole sample (*n* = 8) were no significant. (g) A pair of evoked EPSCs evoked from the cortex in an iSPN in the presence of bicuculline. (h) The decrease in conductance produced by XE991 produces a decrease in EPSCs amplitude as detected at the soma. (i) Superimposition of (g) and (h). (j) Time course of amplitude changes in EPSC. (k) No significant change in the paired pulse ratio (PPR) was detected. (l) Tukey box plots showing that changes in PPR, in the whole sample (*n* = 8), were no significant.

To see whether these effects on the synaptic corticostriatal response of SPNs had a presynaptic component or if all of them had a postsynaptic origin, we evoked pairs of EPSCs in control conditions in the presence of 10 *μ*M bicuculline and these were compared to responses obtained in the presence of XE991. Figures 8(a) and 8(b) show a control recording in a dSPN (average in color and quantal variation in thin grey lines) and a recording in the presence of XE991, respectively. The superimposition is in Figure 8(c). There was a small decrease in current amplitude during the experiment as observed from the soma, suggesting again a decrease in membrane conductance in the region where the synaptic responses are generated (Figure 8(d)); however, there was no change in the paired pulse ratio (PPR; Figure 8(e)). Lack of significance in PPR changes is summarized in Figure 8(f) (*n* = 8). A similar experiment was performed in a sample of iSPNs (Figures 8(g)–8(l)). Here, the decrease in EPSC amplitude was larger suggesting that propagation in iSPNs dendrites is more important than in dSPNs dendrites, given that dendrites form the cell compartment where most synaptic inputs are generated [48, 50]. Nonetheless, changes in PPR were not significant (Figures 8(k) and 8(l)). It was concluded that XE991 reduced EPSCs amplitude in both SPNs without changing PPR and therefore, most actions observed were postsynaptic. In addition, it is known that M_2–4_ receptors, not M_1_ receptors, are located on presynaptic cortical glutamatergic terminals [10–13, 53].

### 3.5. Actions of K_V_7 Channels Minimally Affect the Somatic Compartment

As said before, it is known that most excitatory synapses in SPNs target the dendritic/spines compartment [48, 50, 54–56]. Therefore, the suprathreshold corticostriatal responses described here were most probably generated in the dendritic compartment and recorded in the somatic compartment. But are K_V_7 channels distributed equally in all neuronal membrane? To answer this question we asked what would be the influence of K_V_7 channels in responses evoked at the somatic compartment.

**Figure 9:**
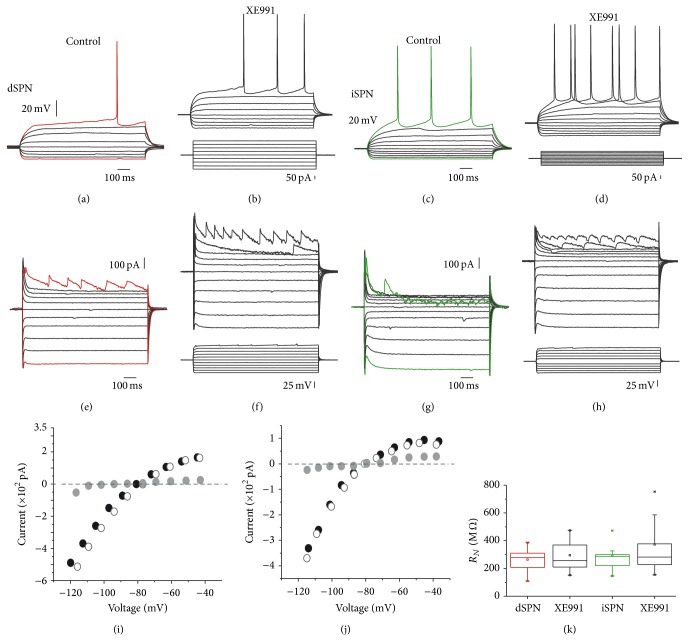
Actions of K_V_7 channels do not occur at the somatic compartment. ((a), (b)) Depolarizations and hyperpolarizations of a dSPN in response to intrasomatic current injections (below (b)) before and after addition of XE991 (20 *μ*M). ((c), (d)) Transmembrane current recordings of the same neurons after depolarizing and hyperpolarizing voltage commands (below (d)). Action currents are clipped. ((e), (f)) Depolarizations and hyperpolarizations of iSPN in response to intrasomatic current injections (below (f)) before and after addition of XE991 (20 *μ*M). ((g), (h)) Transmembrane current recordings of the same neurons after depolarizing and hyperpolarizing voltage commands (below (d)). Action currents are clipped. ((i), (j)) Current-voltage relationships of the dSPN and the iSPN, respectively. Black dots: current measurements in control; white dots: current measurements during XE991; grey dots: subtraction. (k) Tukey box plots illustrate input resistance distributions in samples of dSPNs and iSPNs before and after addition of XE991. Blockade of K_V_7 channels did not have significant actions with these protocols at the somatic compartment.

Figures 9(a)–9(d) show that there were changes in excitability produced in SPNs by XE991 after somatic current injection, although it was more effective at iSPNs. However, actions of Ca^2+^-activated K^+^-currents can also be seen on suprathreshold synaptic responses [26]. Then, point somatic voltage-clamp current-voltage relationships (*I*-*V* plots) were explored in the voltage-clamp mode (Figures 9(e)–9(h); unclamped action currents were clipped). Black dots in Figures 9(i) and 9(j) show control *I*-*V* plots in both classes of dSPNs and iSPNs, respectively, while white dots show the *I*-*V* plots in the same cells after XE991 (10–20 *μ*M). The superposition is almost complete and the subtraction of curves before and after the drug is negligible. In fact, measurements of whole-cell input resistance (*R*_*N*_) in samples of both classes of SPNs had no significant differences (Figure 9(k)) measured at −60 mV. Thus, dSPNs controls had (mean ± SEM) 287 ± 22 MΩ and in the presence of XE991, 265 ± 33 MΩ (*n* = 11; NS); iSPNs had 300 ± 33 MΩ and changed to 381 ± 74 MΩ (*n* = 8; NS).

Thus, although it is well established that dendrites of SPNs express multiple types of potassium channels that contribute to the complexity of neuronal discharge, the functional role of K_V_7 channels during the suprathreshold corticostriatal response of SPNs had not been demonstrated before. Here this functional role is strongly suggested due to the use of selective pharmacological tools. Multiple neurotransmitters have been shown to down- or up-modulate K_V_7 channels [57, 58]. Therefore, this may be a way in which cholinergic innervation controls SPNs firing and circuits. In contrast to these results, K_V_7 channels selectively influence somatic but not dendritic synaptic integration in pyramidal cells from the hippocampus [49, 59–61], although they control synaptic integration in pyramidal cortical neurons [62]. However, in similarity with pyramidal cells, SK-channels also play an important role in SPNs [26] and their interaction needs further study, for example, [63].

**Figure 10:**
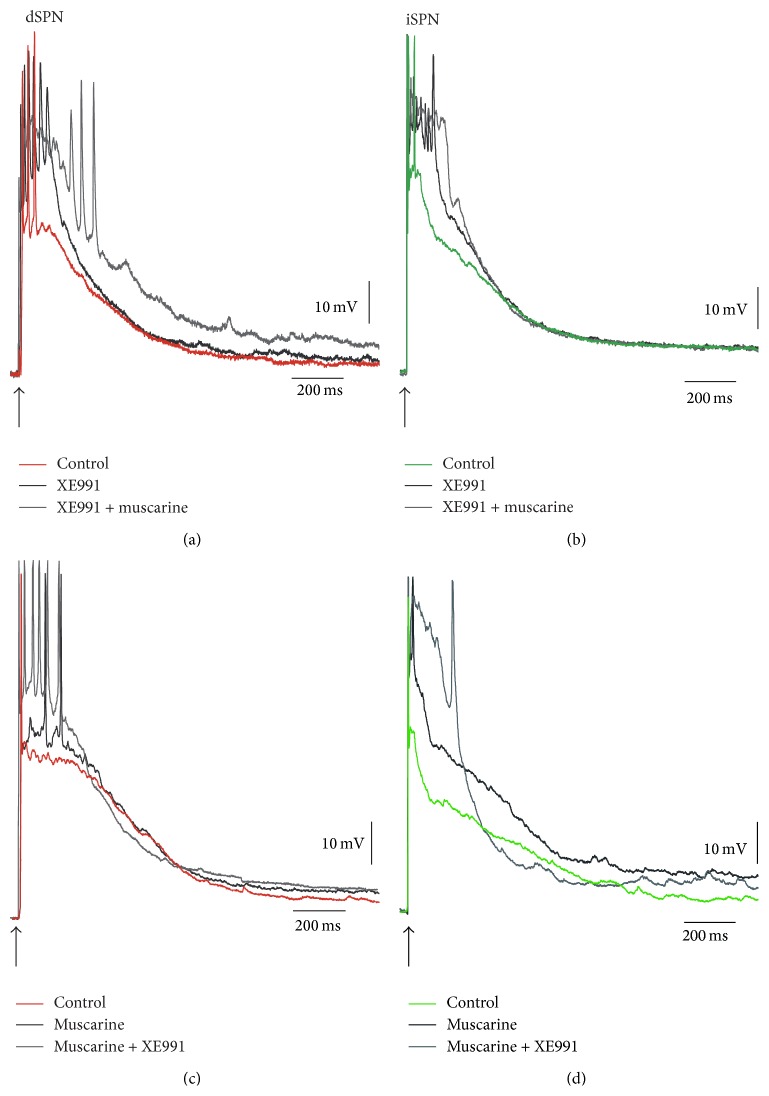
Sequential actions of muscarine and XE991. (a) After XE991 (20 *μ*M) increased control synaptically evoked depolarization in a dSPN, muscarine (1 *μ*M) had an additional action, as expected, due to the presence of M_4_ receptors [24]. (b) This additional action does not happen in iSPNs since they lack M_4_-receptors. (c) After muscarine, XE991 only provoked a small additional depolarization, since it is rare that a receptor action saturates completely a given effector. However, comparing (a) and (c) shows that additional action in (a) suggests that it is not due to M_1_-receptors [24]. (d) Additional action of XE991 is due to an autorregenerative response, which can also appear without it (cf. (b)).

Up to now we have shown that K_V_7 channels may influence synaptic integration because their selective blocker and opener acted on suprathreshold synaptic responses. Also, experiments in Figure 2 showed that a M_1_ muscarinic receptor antagonist and agonist had very similar actions, respectively; they acted similarly in both classes of projection neurons, influenced firing, and their actions lasted during the whole response. Actions such as these had not been observed for other muscarinic effects [24, 26]. To further show these similarities we performed occlusion experiments (Figure 10). However, as mentioned before, muscarinic receptors have many actions on SPNs, on all classes of membrane currents: Na^+^, Ca^2+^, and K^+^ [12, 17–21, 23–26, 30, 34, 44]. Therefore, occlusion experiments cannot be complete. However, if occlusion of these actions is large enough and differences can have a reasonably explanation that could be confirmed experimentally in the future, it could become very suggestive.  Figure 10(a) shows that muscarine (1 *μ*M) had an additional action after XE991 (20 *μ*M), but this does not happen in iSPNs where occlusion is complete (Figure 10(b)). This can be easily explained by the actions of M_4_-receptors in dSPNs which are not present in iSPNs [24]. On the other hand, addition of XE991 in a response of a dSPN that provoked a few action potentials after muscarine obtained only a small additional depolarization. This increased number of action potentials fired shows that actions do not need to be large to influence firing (Figure 10(c)). It is rare that a receptor action saturates any effector, but comparing panels (a) and (c) reinforces the argument of this work and of a previous one [24]. A similar phenomenon occurred in an iSPN whose depolarization did not evoke an autorregenerative response after muscarine but attained it after addition of XE991 (Figure 10(d)). While these experiments (*n* = 6 for each sample) confirmed that muscarinic receptors have various actions on SPNs, the amount of depolarization added to the responses after sequential activation with muscarine and XE991 in either order suggests that a great part of the action may be due to K_V_7 channels.

Since K_V_7 channels are modulated by a variety of neurotransmitters and intracellular signaling molecules [64–66], they provide an exquisite mechanism to fine-tune synaptic convergent integration from the sub- to suprathreshold ranges. It is thought that convergent and prolonged suprathreshold inputs provoke the “down”- to “up”-states voltage transitions [7, 50, 51, 64] characteristic of SPNs firing. It is also known that during these transitions SPNs become involved in correlated firing and network activity such as cell-assembly reverberations [38]. Finally, previous work had shown that a cortical stimulus may provoke prolonged synaptic responses in cholinergic interneurons and SPNs quasisimultaneously [1]. But to respond to released ACh, the receptors expressed by SPNs are G-protein coupled (muscarinic). In particular, modulation of K_V_7 channels involves phosphatidylinositol 4, 5 biphosphate depletion [58, 67–69]. Thus, whether the signaling cascades involved are fast enough to modulate these complex synaptic responses during their whole duration was an open question. In this work we demonstrate that muscarinic signaling is definitively involved in the synaptic integration of SPNs and that this modulation affects the firing of these GABAergic neurons.

## 4. Conclusions

By activating SPNs and cholinergic interneurons almost simultaneously, acetylcholine modulates, through muscarinic receptors, the suprathreshold synaptic integration in striatal projection neurons. In a similar way, K_V_7 channels act as “gain control” regulators of the synaptic response during its whole duration, in both classes of SPNs. Thus, blocking of M_1_ class of muscarinic receptors decreases the responses and greatly abolishes firing, disclosing the action of endogenous acetylcholine [24]: the regulated closing of these channels during the response. Addition of the agonist muscarine does the opposite; it facilitates synaptic depolarization and firing. This last action is potently reproduced by the K_V_7 channel blocker XE991 in both classes of neurons. Conversely, the K_V_7 channel opener, retigabine, mimicked the action of the M_1_ class receptor antagonist; it reduced the responses in both classes of SPNs. It has been demonstrated that muscarinic agonists close K_V_7 channels [34]. Calcium imaging experiments showed that the efficiency of a cortical stimulus to recruit sets of coactive SPNs is increased when K_V_7 channels are closed. Therefore, modulation of these channels not only enhances the response of scattered SPNs but facilitates their working together. In fact, XE991 alone could produce the appearance of “down”- to “up”-state transitions in SPNs. This action was not a random occurrence since it generated a definite increase in microcircuit activity. By inference, we show that the actions of K_V_7 channels are postsynaptic and that they may occur in the dendritic compartment where most synaptic inputs are generated, since their action in somatically evoked responses were minimal. Finally, occlusion was almost complete when XE991 and muscarine are given together in spite of the various muscarinic actions, except when muscarine was given after XE991, suggesting the action of M_4_ receptors [24]. In summary, the present results suggest that antimuscarinic therapy in Parkinson's disease and L-DOPA induced dyskinesia should be more selective and focused on M_1_-class receptors and clinical assays of Parkinsonian-dyskinetic patients using retigabine are scarce. Further basic and clinical research is necessary to better support this suggestion [70].

## References

[1] Vizcarra-Chacón B. J., Arias-García M. A., Pérez-Ramírez M. B. (2013). Contribution of different classes of glutamate receptors in the corticostriatal polysynaptic responses from striatal direct and indirect projection neurons. *BMC Neuroscience*.

[2] Oldenburg I. A., Ding J. B. (2011). Cholinergic modulation of synaptic integration and dendritic excitability in the striatum. *Current Opinion in Neurobiology*.

[3] Pakhotin P., Bracci E. (2007). Cholinergic interneurons control the excitatory input to the striatum. *The Journal of Neuroscience*.

[4] Bonsi P., Cuomo D., Martella G. (2011). Centrality of striatal cholinergic transmission in Basal Ganglia function. *Frontiers in Neuroanatomy*.

[5] Nelson A. B., Bussert T. G., Kreitzer A. C., Seal R. P. (2014). Striatal cholinergic neurotransmission requires VGLUT3. *The Journal of Neuroscience*.

[6] DeBoer P., Westerink B. H. C. (1994). GABAergic modulation of striatal cholinergic interneurons: an in vivo microdialysis study. *Journal of Neurochemistry*.

[7] Stern E. A., Kincaid A. E., Wilson C. J. (1997). Spontaneous subthreshold membrane potential fluctuations and action potential variability of rat corticostriatal and striatal neurons in vivo. *Journal of Neurophysiology*.

[8] Flores-Barrera E., Vizcarra-Chacón B. J., Tapia D., Bargas J., Galarraga E. (2010). Different corticostriatal integration in spiny projection neurons from direct and indirect pathways. *Frontiers in Systems Neuroscience*.

[9] Dodt H. U., Misgeld U. (1986). Muscarinic slow excitation and muscarinic inhibition of synaptic transmission in the rat neostriatum. *The Journal of Physiology*.

[10] Hernández-Echeagaray E., Galarraga E., Bargas J. (1998). 3-*α*-Chloro-imperialine, a potent blocker of cholinergic presynaptic modulation of glutamatergic afferents in the rat neostriatum. *Neuropharmacology*.

[11] Barral J., Galarraga E., Bargas J. (1999). Muscarinic presynaptic inhibition of neostriatal glutamatergic afferents is mediated by Q-type Ca^2+^ channels. *Brain Research Bulletin*.

[12] Galarraga E., Hernández-López S., Reyes A. (1999). Cholinergic modulation of neostriatal output: a functional antagonism between different types of muscarinic receptors. *The Journal of Neuroscience*.

[13] Pancani T., Bolarinwa C., Smith Y., Lindsley C. W., Conn P. J., Xiang Z. (2014). M4 mAChR-mediated modulation of glutamatergic transmission at corticostriatal synapses. *ACS Chemical Neuroscience*.

[14] Hersch S. M., Gutekunst C.-A., Rees H. D., Heilman C. J., Levey A. I. (1994). Distribution of m1-m4 muscarinic receptor proteins in the rat striatum: light and electron microscopic immunocytochemistry using subtype-specific antibodies. *The Journal of Neuroscience*.

[15] Xiang Z., Thompson A. D., Jones C. K., Lindsley C. W., Conn P. J. (2012). Roles of the M1 muscarinic acetylcholine receptor subtype in the regulation of basal ganglia function and implications for the treatment of Parkinson's disease. *The Journal of Pharmacology and Experimental Therapeutics*.

[16] Bernard V., Normand E., Bloch B. (1992). Phenotypical characterization of the rat striatal neurons expressing muscarinic receptor genes. *The Journal of Neuroscience*.

[17] Yan Z., Flores-Hernandez J., Surmeier D. J. (2001). Coordinated expression of muscarinic receptor messenger RNAs in striatal medium spiny neurons. *Neuroscience*.

[18] Pérez-Rosello T., Figueroa A., Salgado H. (2005). Cholinergic control of firing pattern and neurotransmission in rat neostriatal projection neurons: role of CaV2.1 and CaV2.2 Ca^2+^ channels. *Journal of Neurophysiology*.

[19] Shen W., Tian X., Day M. (2007). Cholinergic modulation of Kir2 channels selectively elevates dendritic excitability in striatopallidal neurons. *Nature Neuroscience*.

[20] Akins P. T., Surmeier D. J., Kitai S. T. (1990). Muscarinic modulation of a transient K+ conductance in rat neostriatal neurons. *Nature*.

[21] Carrillo-Reid L., Tecuapetla F., Vautrelle N. (2009). Muscarinic enhancement of persistent sodium current synchronizes striatal medium spiny neurons. *Journal of Neurophysiology*.

[22] Cantrell A. R., Ma J. Y., Scheuer T., Catterall W. A. (1996). Muscarinic modulation of sodium current by activation of protein kinase C in rat hippocampal neurons. *Neuron*.

[23] Pérez-Burgos A., Pérez-Rosello T., Salgado H. (2008). Muscarinic M1 modulation of N and L types of calcium channels is mediated by protein kinase C in neostriatal neurons. *Neuroscience*.

[24] Hernández-Flores T., Hernández-Gonzáles O., Pérez-Burgos A. (2015). Modulation of direct pathway striatal projection neurons by muscarinic M4-type receptors. *Neuropharmacology*.

[25] Howe A. R., Surmeier D. J. (1995). Muscarinic receptors modulate N-, P-, and L-type Ca^2+^ currents in rat striatal neurons through parallel pathways. *The Journal of Neuroscience*.

[26] Arias-García M. A., Tapia D., Flores-Barrera E., Pérez-Ortega J. E., Bargas J., Galarraga E. (2013). Duration differences of corticostriatal responses in striatal projection neurons depend on calcium activated potassium currents. *Frontiers in Systems Neuroscience*.

[27] Pisani A., Bernardi G., Ding J., Surmeier D. J. (2007). Re-emergence of striatal cholinergic interneurons in movement disorders. *Trends in Neurosciences*.

[28] Plotkin J. L., Day M., Surmeier D. J. (2011). Synaptically driven state transitions in distal dendrites of striatal spiny neurons. *Nature Neuroscience*.

[29] Benarroch E. E. (2012). Effects of acetylcholine in the striatum: recent insights and therapeutic implications. *Neurology*.

[30] Goldberg J. A., Ding J. B., Surmeier D. J. (2012). Muscarinic modulation of striatal function and circuitry. *Muscarinic Receptors*.

[31] Wang W., Darvas M., Storey G. P. (2013). Acetylcholine encodes long-lasting presynaptic plasticity at glutamatergic synapses in the dorsal striatum after repeated amphetamine exposure. *The Journal of Neuroscience*.

[32] Adams P. R., Brown D. A. (1982). Synaptic inhibition of the M-current: slow excitatory post-synaptic potential mechanism in bullfrog sympathetic neurones. *The Journal of Physiology*.

[33] Brown D. A., Adams P. R. (1980). Muscarinic suppression of a novel voltage-sensitive K^+^ current in a vertebrate neurone. *Nature*.

[34] Shen W., Hamilton S. E., Nathanson N. M., Surmeier D. J. (2005). Cholinergic suppression of KCNQ channel currents enhances excitability of striatal medium spiny neurons. *The Journal of Neuroscience*.

[35] Soh H., Pant R., LoTurco J. J., Tzingounis A. V. (2014). Conditional deletions of epilepsy-associated KCNQ2 and KCNQ3 channels from cerebral cortex cause differential effects on neuronal excitability. *The Journal of Neuroscience*.

[36] Wu W. W., Chan C. S., Surmeier D. J., Disterhoft J. F. (2008). Coupling of L-type Ca^2+^ channels to K_V_7/KCNQ channels creates a novel, activity-dependent, homeostatic intrinsic plasticity. *Journal of Neurophysiology*.

[37] English D. F., Ibanez-Sandoval O., Stark E. (2012). GABAergic circuits mediate the reinforcement-related signals of striatal cholinergic interneurons. *Nature Neuroscience*.

[38] Carrillo-Reid L., Tecuapetla F., Tapia D. (2008). Encoding network states by striatal cell assemblies. *Journal of Neurophysiology*.

[39] Contant C., Umbriaco D., García S., Watkins K. C., Descarries L. (1996). Ultrastructural characterization of the acetylcholine innervation in adult rat neostriatum. *Neuroscience*.

[40] Wilson C. J., Goldberg J. A. (2006). Origin of the slow afterhyperpolarization and slow rhythmic bursting in striatal cholinergic interneurons. *Journal of Neurophysiology*.

[41] Zhou F.-M., Wilson C. J., Dani J. A. (2002). Cholinergic interneuron characteristics and nicotinic properties in the striatum. *Journal of Neurobiology*.

[42] Lim S. A., Kang U. J., McGehee D. S. (2014). Striatal cholinergic interneuron regulation and circuit effects. *Frontiers in Synaptic Neuroscience*.

[43] Goldberg J. A., Teagarden M. A., Foehring R. C., Wilson C. J. (2009). Nonequilibrium calcium dynamics regulate the autonomous firing pattern of rat striatal cholinergic interneurons. *The Journal of Neuroscience*.

[44] Hsu K. S., Yang C. H., Huang C. C., Gean P. W. (1996). Carbachol induces inward current in neostriatal neurons through M1-like muscarinic receptors. *Neuroscience*.

[45] Bennett B. D., Callaway J. C., Wilson C. J. (2000). Intrinsic membrane properties underlying spontaneous tonic firing in neostriatal cholinergic interneurons. *The Journal of Neuroscience*.

[46] Jerusalinsky D., Harvey A. L. (1994). Toxins from mamba venoms: small proteins with selectivities for different subtypes of muscarinic acetylcholine receptors. *Trends in Pharmacological Sciences*.

[47] Bradley K. N. (2000). Muscarinic toxins from the green mamba. *Pharmacology and Therapeutics*.

[48] Plotkin J. L., Shen W., Rafalovich I. (2013). Regulation of dendritic calcium release in striatal spiny projection neurons. *Journal of Neurophysiology*.

[49] Hu H., Vervaeke K., Storm J. F. (2007). M-channels (Kv7/KCNQ channels) that regulate synaptic integration, excitability, and spike pattern of CA1 pyramidal cells are located in the perisomatic region. *The Journal of Neuroscience*.

[50] Day M., Wokosin D., Plotkin J. L., Tian X., Surmeier D. J. (2008). Differential excitability and modulation of striatal medium spiny neuron dendrites. *The Journal of Neuroscience*.

[51] Vergara R., Rick C., Hernández-López S. (2003). Spontaneous voltage oscillations in striatal projection neurons in a rat corticostriatal slice. *The Journal of Physiology*.

[52] Leão R. N., Tan H. M., Fisahn A. (2009). Kv7/KCNQ channels control action potential phasing of pyramidal neurons during hippocampal gamma oscillations in vitro. *The Journal of Neuroscience*.

[53] Malenka R. C., Kocsis J. D. (1988). Presynaptic actions of carbachol and adenosine on corticostriatal synaptic transmission studied in vitro. *The Journal of Neuroscience*.

[54] Wilson C. J., Groves P. M. (1980). Fine structure and synaptic connections of the common spiny neuron of the rat neostriatum: a study employing intracellular injection of horseradish peroxidase. *The Journal of Comparative Neurology*.

[55] Xu Z. C., Wilson C. J., Emson P. C. (1989). Restoration of the corticostriatal projection in rat neostriatal grafts: electron microscopic analysis. *Neuroscience*.

[56] Carter A. G., Sabatini B. L. (2004). State-dependent calcium signaling in dendritic spines of striatal medium spiny neurons. *Neuron*.

[57] Brown B. S., Yu S. P. (2000). Modulation and genetic identification of the M channel. *Progress in Biophysics and Molecular Biology*.

[58] Marrion N. V. (1997). Control of M-current. *Annual Review of Physiology*.

[59] Yue C., Yaari Y. (2006). Axo-somatic and apical dendritic Kv7/M channels differentially regulate the intrinsic excitability of adult rat CA1 pyramidal cells. *Journal of Neurophysiology*.

[60] Shah M. M., Migliore M., Valencia I., Cooper E. C., Brown D. A. (2008). Functional significance of axonal Kv7 channels in hippocampal pyramidal neurons. *Proceedings of the National Academy of Sciences of the United States of America*.

[61] Shah M. M., Migliore M., Brown D. A. (2011). Differential effects of Kv7 (M-) channels on synaptic integration in distinct subcellular compartments of rat hippocampal pyramidal neurons. *The Journal of Physiology*.

[62] Lee S., Kwag J. (2012). M-channels modulate the intrinsic excitability and synaptic responses of layer 2/3 pyramidal neurons in auditory cortex. *Biochemical and Biophysical Research Communications*.

[63] Mateos-Aparicio P., Murphy R., Storm J. F. (2014). Complementary functions of SK and Kv7/M potassium channels in excitability control and synaptic integration in rat hippocampal dentate granule cells. *The Journal of Physiology*.

[64] Delmas P., Brown D. A. (2005). Pathways modulating neural KCNQ/M (Kv7) potassium channels. *Nature Reviews Neuroscience*.

[65] Soldovieri M. V., Miceli F., Taglialatela M. (2011). Driving with no brakes: molecular pathophysiology of K_v_7 potassium channels. *Physiology*.

[66] Gamper N., Zaika O., Li Y. (2006). Oxidative modification of M-type K^+^ channels as a mechanism of cytoprotective neuronal silencing. *The EMBO Journal*.

[67] Suh B. C., Hille B. (2002). Recovery from muscarinic modulation of M current channels requires phosphatidylinositol 4,5-bisphosphate synthesis. *Neuron*.

[68] Suh B.-C., Inoue T., Meyer T., Hille B. (2006). Rapid chemically induced changes of PtdIns(4,5)P_2_ gate KCNQ ion channels. *Science*.

[69] Zaydman M. A., Cui J. (2014). PIP2 regulation of KCNQ channels: biophysical and molecular mechanisms for lipid modulation of voltage-dependent gating. *Frontiers in Physiology*.

[70] Sander S. E., Lemm C., Lange N., Hamann M., Richter A. (2012). Retigabine, a K(V)7 (KCNQ) potassium channel opener, attenuates L-DOPA-induced dyskinesias in 6-OHDA-lesioned rats. *Neuropharmacology*.

